# PYK2 mediates the BRAF inhibitor (vermurafenib)-induced invadopodia formation and metastasis in melanomas

**DOI:** 10.20892/j.issn.2095-3941.2020.0294

**Published:** 2021-09-28

**Authors:** Junling Shen, Jilong Yang, Lei Sang, Rui Sun, Weiyu Bai, Chao Wang, Yan Sun, Jianwei Sun

**Affiliations:** 1 Center for Life Sciences, School of Life Sciences, State Key Laboratory for Conservation and Utilization of Bio-Resources in Yunnan, Yunnan University, Kunming 650091, China; 2 Tianjin Medical University Cancer Institute and Hospital, National Clinical Research Center for Cancer, Key Laboratory of Cancer Prevention and Therapy, Tianjin’s Clinical Research Center for Cancer, Tianjin, China

**Keywords:** STIM1, Pyk2, invadopodia, invasion, melanoma, vemurafenib

## Abstract

**Objective:**

The BRAF inhibitor, vemurafenib, has been widely used in the treatment of patients with melanoma-bearing BRAF^V600E^ mutations. While the initial response to vemurafenib is usually excellent, the majority of patients eventually develop resistance and metastatic disease. However, the underlying molecular mechanism remains elusive. The objective of this study was therefore to identify additional molecular targets responsible for vemurafenib resistance.

**Methods:**

Western blots and immunohistochemistry analyses were used to evaluate expressions of PYK2 and p-PYK2 in cultured cells and melanoma tissue microarrays. The relationships of p-PYK2 with clinicopathological parameters were statistically analyzed. Invadopodia cell invasion, and a Ca^2+^ assay were used to determine the effect of vemurafenib resistance-induced p-PYK2 on melanoma progression. A mouse model was used to assess the effects of PYK2 on melanoma metastasis.

**Results:**

Elevated p-PYK2 levels were detected in vemurafenib-resistant melanoma cells, and PYK2 was shown to regulate invadopodia formation in melanoma cells. Vemurafenib triggered invadopodia formation by activation of PYK2. Inhibition of PYK2 with either shRNA or the small molecule inhibitor, PF562711, dramatically reduced vemurafenib-induced invadopodia formation. Furthermore, knockdown of PYK2 significantly reduced melanoma lung metastasis *in vivo*. Increased expressions of p-PYK2 in melanoma patients were positively correlated with advanced stage (*P* = 0.002), metastasis (*P* < 0.001), and Clark grade (*P* < 0.001), and were also associated with short overall survival [hazard ratio (HR) = 3.304, *P* = 0.007] and progression-free survival (HR = 2.930, *P* = 0.001).

**Conclusions:**

PYK2 mediated vemurafenib-induced melanoma cell migration and invasion. Inhibition of PYK2 resensitized melanoma cells to vemurafenib. Phospho-PYK2 was a prognostic biomarker in melanoma patients.

## Introduction

Melanoma represents 10% of all skin cancers and is responsible for 80% of skin cancer-related deaths^1^. The 5 year survival for early stage melanoma treated by surgical resection is as high as 98%^2^; however, for advanced stage melanoma, the 5 year survival is only 16%^3^. An alkylating agent, dacarbazin (DTIC), has been the traditional treatment for patients with advanced stage melanoma. However, DTIC treatment in melanoma patients has only a 10% response, with serious side effects and 1 year survival of only 36%^4^. The discovery of mutant specific BRAF inhibitors, such as vemurafenib, has remarkably improved the survival of melanoma patients with oncogenic BRAF mutations. However, vemurafenib resistance occurred in approximately 50% of the patients, and approximately half of the patients had disease progression within 6 months from the start of vemurafenib monotherapy, which greatly hindered the clinical effectiveness of vemurafenib^5^. Identification of the mechanism by which melanoma resists vemurafenib will therefore provide potential target(s) for vemurafenib combination therapy^6–8^. Recent studies have shown the reactivation of the MAPK pathway in vemurafenib-resistant melanomas. In addition, accumulating evidence has indicated that vemurafenib treatment induced selective pressure to promote melanoma cell migration, invasion, and metastasis^9,10^. However, the mechanism by which vemurafenib induced melanoma invasion and metastasis is still not completely understood.

Invadopodia are specialized, actin-rich membrane protrusions mediating focal extracellular matrix (ECM) degradation in malignant cancer cells^11–13^. Invadopodia assembly is initiated in response to the focal generation of phosphatidylinositol-3,4-biphosphate and activation of the non-receptor tyrosine kinase, Src, which recruits adaptor protein TKS5 and cortactin to assemble the actin core of invadopodium^14–17^. Upon maturation, invadopodia recruit and secrete proteinases such as membrane type 1-matrix metalloproteinase (MMP), MMP2, and MMP9 to degrade the ECM and facilitate invasion^15–17^. The proline rich tyrosine kinase 2 (PYK2, also termed FAK2), a calcium-dependent focal adhesion kinase, has been reported to be involved in tumorigenesis^18,19^. A recent study reported that PYK2 played a pivotal role in invadopodia formation in breast cancer^20^. However, the role of PYK2 in melanoma invasion is not clear.

Here we showed that Pyk2 activation was crucial for invadopodia formation and cell invasion in melanomas. The BRAF inhibitor, vermurafenib, induced invadopodia formation and promoted cell invasion at least partly through activation of PYK2. The PYK2 inhibitor, PF562271, decreased invadopodia formation and cell invasion induced by vemurafenib in melanoma cells. In addition, the p-PYK2 level was positively correlated with higher Clark grade, advanced stage, metastasis, as well as poor overall survival (OS) and progress-free survival (PFS) in melanoma patients. The present study demonstrated a role for PYK2 in melanoma progression, and provided a potential combination-therapy strategy to improve the therapeutic efficiency of BRAF inhibitors in melanoma patients.

## Materials and methods

### Cell culture and inhibitor treatment

Melanoma cell lines, WM793 and 1205Lu, were cultured in RPMI 1640 medium supplemented with 10% fetal bovine serum (FBS) and 1% penicillin/streptomycin. Vemurafenib resistant WM793 cells were a generous gift from Dr. Keiran Smalley (H. Lee Moffitt Cancer Center, Tampa, FL, USA) and were maintained in RPMI 1640 medium supplemented with 5% FBS and 1 µM vemurafenib^9^. For inhibitor treatment, cells (5 × 10^5^) were seeded in 6-well plates and treated with 2 µM vemurafenib or PF562711 for 12 h, 24 h, and 36 h after the cells attached to the dish.

### RNA interference

RNA interference of PYK2 was performed using the pSUPER.Retro.puro vector (Oligoengine, Seattle, WA, USA). To efficiently knockdown PYK2, 2 shRNAs targeting 2 different regions of the PYK2 gene were used. The target sequences were TGCACTTGACAAGAAGTCC (PYK2 sh1) and ACCCAGAAACTGCTCAAC (PYK2 sh2).

### Antibodies

The following antibodies were used in this study: rabbit polyclonal anti-PYK2 (3292; Cell Signaling Technology, Danvers, MA, USA), rabbit polyclonal anti-p-PYK2 (Y402) (3291; Cell Signaling Technology), mouse monoclonal anti-p-PYK2 (Y402) (sc-293142; Santa Cruz Biotechnology, Santa Cruz, CA, USA), rabbit polyclonal anti-FAK (3285; Cell Signaling Technology), rabbit polyclonal anti-p-FAK (Tyr397) (3283; Cell Signaling Technology), and mouse monoclonal anti-glyceraldehyde 3-phosphate dehydrogenase (GAPDH) (G8795; Sigma-Aldrich, St. Louis, MO, USA).

### Western blot analysis

Western blotting was performed as previously described^21^. Briefly, 50 µg of protein in whole cell lysates from each sample were loaded on a 10% PAGE gel; the membrane was blocked in 5% nonfat milk in 1× Tris-buffered saline (pH 7.4) containing 0.05% Tween-20, then probed with primary antibodies at a concentration of 1:1,000 (anti-PYK2, anti-p-PYK2, anti-FAK, and anti-p-FAK) or 1:5,000 (anti-GAPDH). The secondary antibodies were used at a concentration of 1:10,000 to 1:20,000. The proteins were visualized with the ECL-plus western blotting detection system (5200Multi; Tanon Science and Technology, Shanghai, China).

### Immunofluorescence staining

Cells (8 × 10^4^) were seeded onto glass coverslips coated with 0.2% gelatin and cultured in complete medium under standard cell culture conditions for 4 h. The cells were fixed in 4% paraformaldehyde at ambient temperature for 15 min, followed by permeabilization in 1× phosphate-buffered saline (PBS) containing 0.05% NP-40 for 30 min at room temperature (RT). The cells were then blocked in a blocking solution (1× PBS containing 10% normal goat serum and 0.05% NP-40) for at least 4 h. After washing with 1× PBS, the cells were incubated with rabbit polyclonal anti-p-PYK2-Y402 antibody (1:1,000 dilution in the blocking solution) for 1 h at RT. Following washing, the cells were incubated with a goat anti-rabbit IgG conjugated with Alexa Fluor 488 (A11029; Invitrogen, Carlsbad, CA, USA) (1:1,000 in the blocking solution) at RT for 1 h. After phalloidin staining using phalloidin-TRITC (Molecular Probes, Invitrogen), fluorescence images were obtained with an upright fluorescence microscope (Axio Observer.Z1; Carl Zeiss, Jena, Germany).

### Invadopodia assay

The invadopodia assay was conducted as previously described^21–23^. Briefly, WM793 cells (8 × 10^4^) were plated onto glass coverslips coated with gelatin and allowed to attach for 4 h in a CO_2_ incubator at 37 °C. The cells were then fixed with fresh 4% paraformaldehyde, permeabilized in an antibody diluting buffer (2% bovine serum albumin and 0.1% Triton X-100 in PBS), and subsequently incubated with Alexa Fluor 488-labeled phalloidin (1:100 dilution from a 20 U/mL stock solution) for 30 min. The coverslips were then mounted onto slides in mounting medium (150 mM Tris, pH 8.0, and 90% glycerol). Invadopodia were visualized with an upright fluorescence microscope (Axio Observer.Z1; Carl Zeiss).

### Invasion assay

The cell invasion assay was conducted using BD Matrigel-coated invasion chambers (6.5 mm diameter, 8 µm pore size; BD Biosciences, San Jose, CA, USA)^22^. WM793 cells (1 × 10^5^) suspended in serum-free medium were plated in the upper chamber of the invasion insert. After incubation for 12 h, the cells were fixed with 3.7% formaldehyde and stained with Crystal Violet solution. Three randomly selected fields (10× objective) on the lower side of the insert were photographed, and the cells on the lower surface of the insert were counted. Vemurafenib resistant WM793 cells were cultured in RPMI 1640 medium containing 1 µM of vemurafenib. Vemurafenib was removed from the medium 2 days before the invasion assay.

### Cell death and cell cycle assays

After treatment with 2 µM vemurafenib for 48 h, WM793 cells were analyzed by flow cytometry to determine cell death and the cell cycle^24^. Briefly, cells were trypsinized and then washed with cold PBS twice. Following fixation with cold 70% ethanol overnight and washing with ice cold PBS, the cells were incubated in propidium iodide/RNase staining buffer (C1052; Beyotime, Beijing, China) for 30 min and then analyzed using flow cytometry.

### Patients and data collection

After approval by the Institutional Review Board (bc2021105), 180 formalin-fixed, paraffin-embedded melanoma tissues were collected at the Department of Pathology at Tianjin Medical University Cancer Institute and Hospital. All samples were from patients who underwent surgery from June 2007 to February 2013. None of the patients received chemotherapy or radiotherapy before surgery. Data on age, sex, anatomical site of the primary tumor, clinical stage at diagnosis, therapy, and follow-up were obtained from the Records Office at Tianjin Medical University Cancer Institute and Hospital.

### Tissue microarray and immunohistochemistry

Tissue microarrays (TMAs) were constructed using a manual tissue microarray instrument (Beecher Instruments, Sun Prairie, WI, USA) equipped with a 2.0 mm punch needle, as previously described^25^. TMAs were immunohistochemically stained with mouse monoclonal anti-human p-PYK2-Y402 antibody (1:200, Santa Cruz Biotechnology, Santa Cruz, CA, USA). The positive cells for p-PYK2 were defined as those with brown staining. The positive ratio of p-PYK2 was defined as the percent of cells positive for p-PYK2 staining in melanoma cells. Using the median positive ratio of p-PYK2 as the cut-off value, patients were divided into either high or low p-PYK2 expression groups.

### Statistical analysis

All experiments were repeated 3 times, and each experiment involved 3 replicates per sample. Data were analyzed using SPSS statistical software for Windows, version 19.0 (SPSS, Chicago, IL, USA) and Prism 6.0 software (GraphPad, San Diego, CA, USA). Student’s *t*-test, Spearman’s correlation, Kaplan-Meier log-rank test, and Cox regression survival were used, and statistical significances were defined as *P < 0.05, **P < 0.01, or ***P < 0.001.

## Results

### Vemurafenib resistant melanoma cells exhibit more invadopodia formation

**Figure 1:**
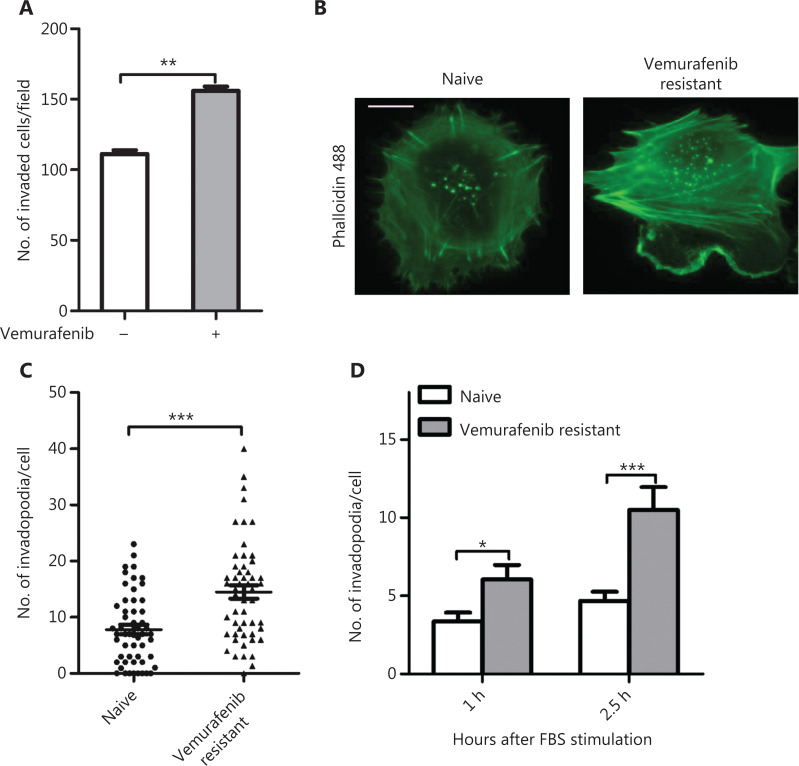
Vermurafenib resistant melanoma cells exhibited more invasiveness and invadopodia formation. (A) Quantitation of the invaded cells in vemurafenib resistant WM793 cells cultured with and without vemurafenib (*n* = 3). (B, C) Representative images of actin fluorescence staining showing invadopodia (B) and quantification of invadopodia (C) in naïve and vermurafenib resistant WM793 cells (*n* = 3). (D) Quantitation of invadopodia at indicated times after 10% fetal bovine serum stimulation in naive and vermurafenib resistant WM793 cells (*n* = 3). Scale bars = 10 μm. *P < 0.05; **P < 0.01; ***P < 0.001.

**Figure 2:**
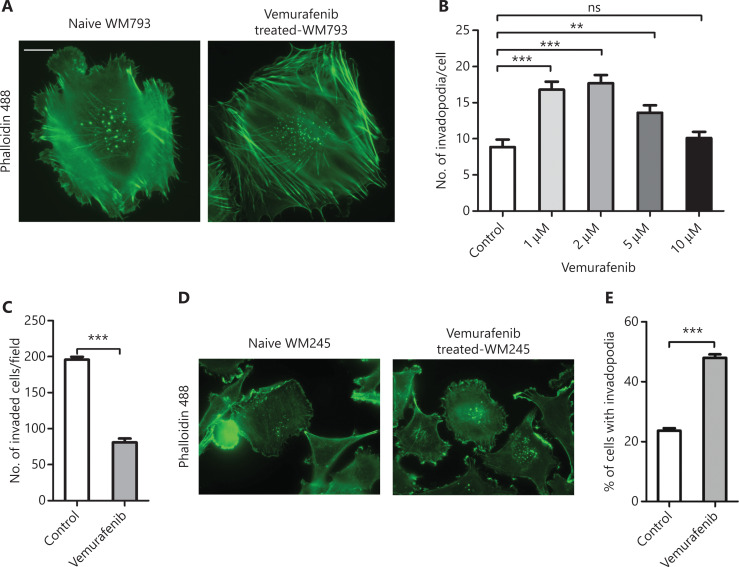
Vemurafenib triggers the formation of invadopodia. (A) Representative images of actin fluorescence staining showing invadopodia in WM793 cells. Scale bars = 10 μm. (B) Quantitation of invadopodia in WM793 cells treated with different concentrations of vemurafenib. (C) Quantitation of invaded WM793 cells treated with and without vemurafenib. (D) Representative images of actin fluorescence staining of invadopodia in WM245 cells. (E) Quantitation of WM245 cells with invadopodia, treated with and without vemurafenib (*n* = 3). **P < 0.01; ***P < 0.001.

Studies showed that vemurafenib paradoxically promoted melanoma invasion and metastasis in vemurafenib resistant melanomas^9,10^. To understand the underlying molecular mechanism, invasion assays were performed using vemurafenib resistant WM793 cells. We found that the invasion ability of resistant cells was significantly reduced after removing vemurafenib from the medium (**Figure 1A**). We next examined invadopodia formation in naïve and vemurafenib resistant WM793 cells. The results showed that the vemurafenib resistant cells had more invadopodia (**Figure 1B and 1C**). To determine the *de novo* invadopodia formation in these cells, the naïve and resistant cells were starved in medium containing 1% FBS overnight and then stimulated with 10% FBS to induce *de novo* assembly of invadopodia. **Figure 1D** shows that vemurafenib resistant cells assembled more *de novo* invadopodia than naïve cells. To examine whether vemurafenib treatment induced invadopodia formation, we first treated WM793 cells with different concentrations of vemurafenib for 48 h and then conducted the invadopodia assay. Although vemuarfenib treatment significantly inhibited cell invasion in naïve WM793 cells (**Figure 2C**), there was more invadopodia formation in the low concentration vemurafenib-treated cells when compared to the untreated naïve WM793 cells (**Figure 2A and 2B**). Notably, vemurafenib-induced invadopodia formation was dose-independent, which could be due to cell toxicity of high doses of vemurafenib (**Figure 2B**). To test this possibility, WM245 cells were treated with 2 µM vemurafenib for 48 h and then subjected to the invadopodia assay. The results showed that the percentage of WM245 cells with invadopodia increased from 20% to more than 40% after vemurafenib treatment (**Figure 2D and 2E**).

We also found that vemurafenib treatment significantly inhibited two-dimensional (2D) and three-dimensional (3D) cell colony formation in naïve WM793 cells, while vemurafenib treatment had little effect on colony formation and soft agar colony growth in vemurafenib resistant WM793 cells, suggesting that the resistant cells had a stronger survival ability and tumorigenic potential during vemurafenib stress (**Supplementary Figure S1**), which possibly resulted from vemurafenib-induced cell death and cell growth arrest in naïve cells.

### The p-PYK2 is necessary for invadopodia formation and melanoma cell invasion

**Figure 3:**
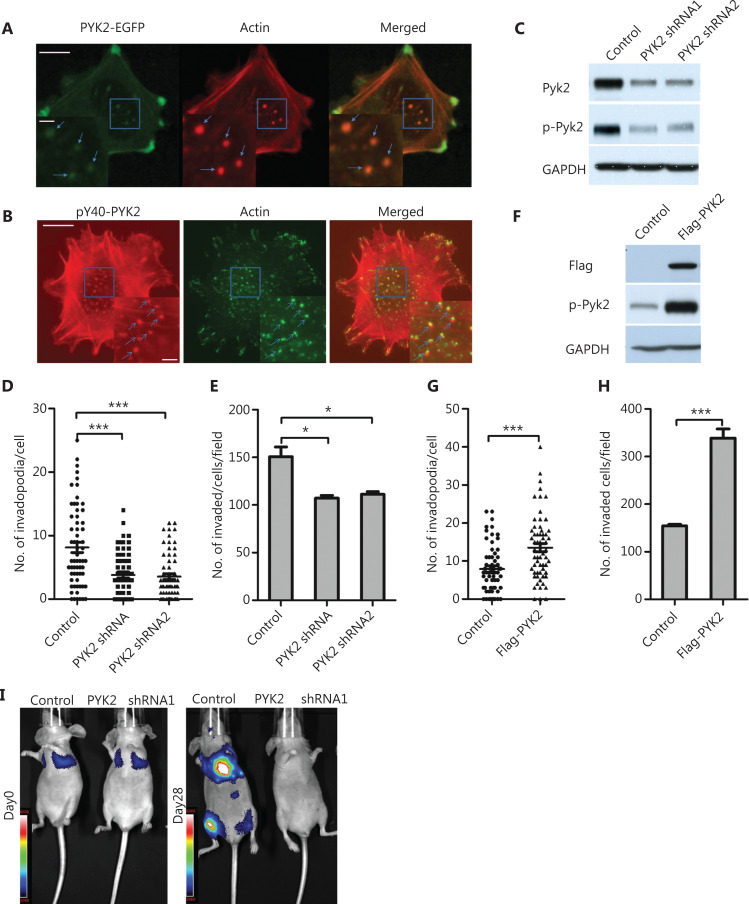
Phospho-PYK2-Y402 localizes at invadopodia and regulates invadopodia formation and cell invasion in naïve WM793 cells. (A) Representative images of fluorescence staining shows that PYK2-EGFP was localized at invadopodia (actin staining). (B) Representative images of fluorescence staining show p-PYK2 localized at invadopoida. (C–E) Western blot analysis of expressions of PYK2 and p-PYK2 (C), quantification of invadopodia (D) and invaded cells (E) in PYK2-knockdown and control WM793 cells (*n* = 3). (F–H) Western blot analyses of PYK2 and p-PYK2 expressions in PYK2-transfected and control WM793 cells (F), and quantification of invadopodia (G) and invaded cells (H) in PYK2-overexpressing and control WM793 cell (*n* = 3). (I) Knockdown of PYK2 in 1205Lu cells significantly reduced lung metastases in a mouse model. Scale bars = 10 μm in the main images; Scale bars = 2 μm in the inserts. *P < 0.05; ***P < 0.001.

Previous studies showed that PYK2 was crucial for invadopodia formation in breast cancer^20^. We therefore determined the role of PYK2 in vemurafenib-induced invadopodia formation and cell invasion. Immunofluorescence staining of naïve WM793 cells showed that PYK2-EGFP co-localized with actin-stained invadopodia (**Figure 3A**), and that p-PYK2-Y402 predominantly localized to invadopodia (**Figure 3A and 3B**). Depletion of PYK2 in WM793 cells dramatically decreased the number of invadopodia and inhibited cell invasion (**Figure 3C, E**). Conversely, overexpression of PYK2 increased invadopodia formation and cell invasion (**Figure 3F, H**). Together, these results indicated that PYK2 was critical for invadopodia formation and cell invasion in melanomas.

To define the role of PYK2 in melanoma metastasis in an animal model, we knocked-down PYK2 in 1205Lu melanoma cells in which luciferase had been stably transfected. The 1205Lu cells treated with PYK2-shRNA or control shRNA were injected into the tail vein of nude mice, and lung metastasis was monitored using bioluminescence imaging. The results showed that knockdown of PYK2 significantly reduced lung metastasis as compared to control shRNA-treated 1205 Lu cells (**Figure 3I**), suggesting that PYK2 played a critical in melanoma metastasis.

### The p-PYK2 is required for vemurafenib-induced invadopodia formation

**Figure 4:**
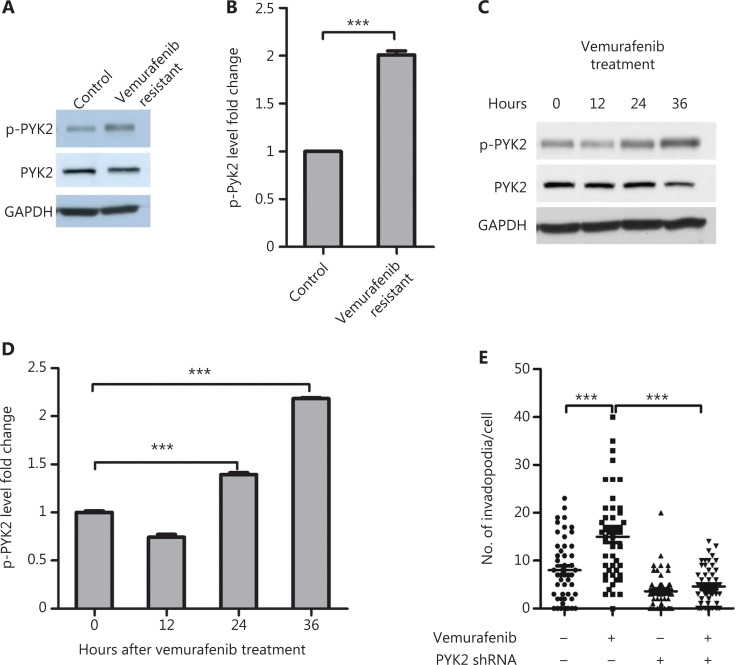
P-PYK2 mediates vemurafenib-induced invadopodia formation in vemurafenib resistant WM793 cells. (A, B) Western blot analyses (A) and quantification of p-PYK2 levels (B) in naïve and vemurafenib resistant WM793 cells (*n* = 3). (C, D) Western blot analyses (C) and the quantification (D) of p-PYK2 levels in WM793 cells treated with and without vemurafenib at the indicated times (*n* = 3). (E) Quantification of invadopodia in PYK2-knockdown and control cells treated with and without vemurafenib (*n* = 3). ***P < 0.001.

We next investigated whether PYK2 was responsible for invadopodia formation in vemurafenib resistant melanoma cells. Western blot analysis showed that the level of p-PYK2-Y402 in resistant cells was 2-fold higher than that in naïve cells (**Figure 4A and 4B**). Furthermore, vemurafenib treatment of naïve cells increased p-PYK2-Y402 levels in naïve WM793 cells after treatment for 24 and 36 h (**Figure 4C and 4D**).

To further examine whether PYK2 activation mediated vemurafenib-induced invadopodia formation, PYK2 was knocked-down with shRNA in vemurafenib resistant WM793 cells. We found that depletion of PYK2 significantly inhibited invadopodia formation induced by vemurafenib treatment (**Figure 4E**). These results indicated that PYK2 kinase was activated by vemurafenib and also played a pivotal role in vemurafenib-induced invadopodia formation in vemurafenib resistant melanomas.

### Targeting PYK2 inhibits vemurafenib-induced invadopodia formation and melanoma cell invasion

**Figure 5:**
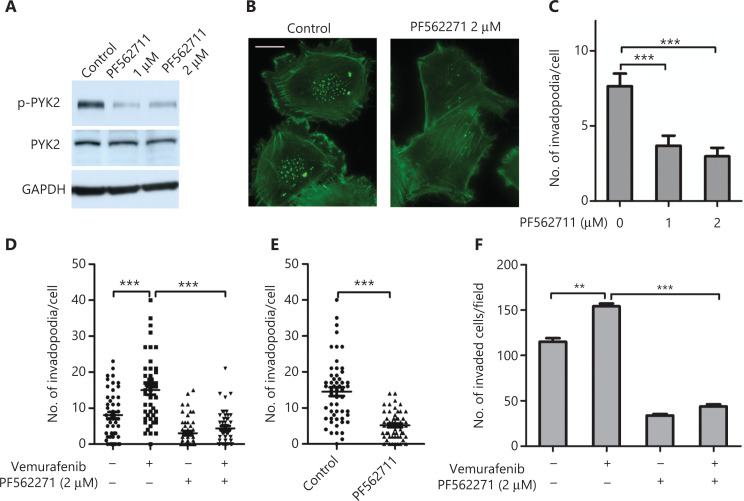
The PYK2 inhibitor, PF562711, abrogates vemurafenib-induced invadopodia formation and cell invasion. (A–C) Western blot analysis of p-PYK2 levels (A), representative images of fluorescence staining of actin (B), and quantification of invadopodia (C) in WM793 cells treated with dimethyl sulfoxide or PF562711 (*n* = 3). (D) Quantification of invadopodia in WM793 cells treated with and without vemurafenib and/or PF562711 (*n* = 3). (E, F) Quantification of invadopodia (E) and invaded cells (F) in vemurafenib resistant WM793 cells treated with and without vemurafenib and/or PF562711 (*n* = 3). Scale bars = 10 μm. **P < 0.01; ***P < 0.001.

Because vemurafenib-induced invadopodia was regulated by PYK2, we next investigated if the PYK2 inhibitor, PF562711, decreased vemurafenib-induced invadopodia. **Figure 5** shows that PF562711 treatment dramatically inhibited p-PYK2 levels (**Figure 5A**) and invadopodia formation in WM793 cells (**Figure 5B and 5C**). Notably, treatment of vemurafenib resistant WM793 cells with PF562711 inhibited vemurafenib-induced invadopodia formation (**Figure 5D and 5E**) and cell invasion (**Figure 5F**). These findings suggested that the PYK2 inhibitor, PF562711, may be a potential therapeutic agent to override vemurafenib-induced cell invasion and metastasis in vemurafenib resistant melanomas.

### Vemurafenib induces PYK2 activation and invadopodia formation through the STIM1-Ca^2+^/CAM axis

**Figure 6:**
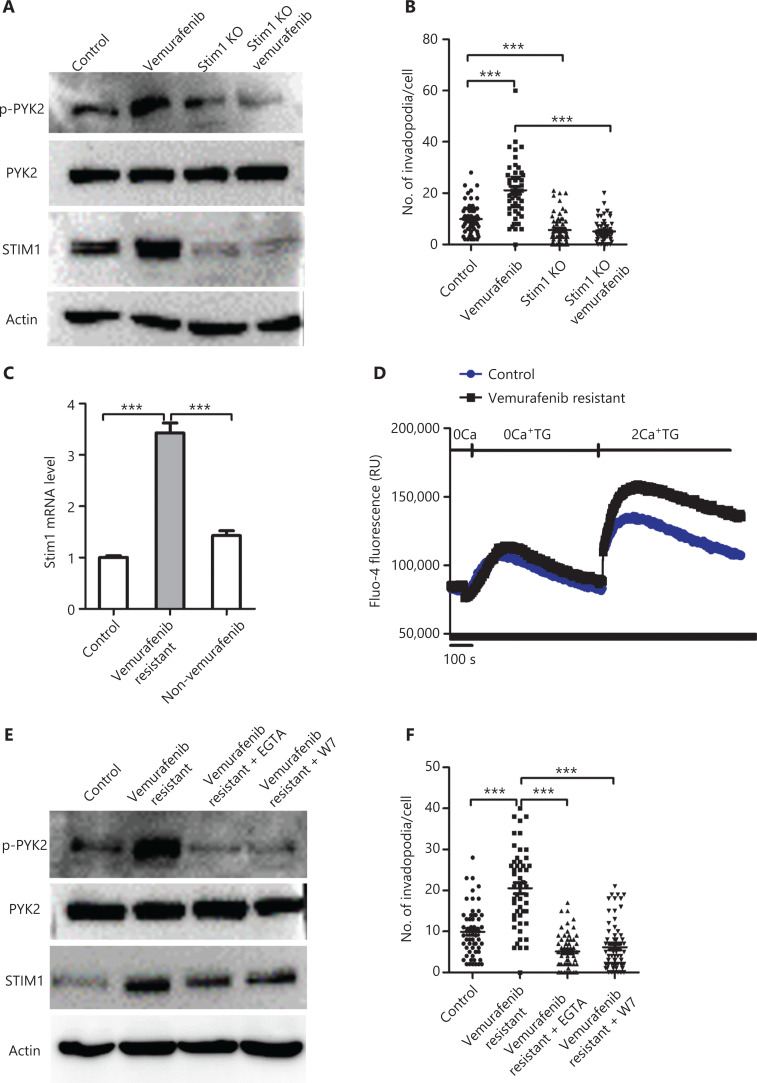
STIM1 mediates vemurafenib-induced PYK2 activation and invadopodia formation. (A) Western blot analysis of p-PYK2 and STIM1 expressions in STIM1 knockout (KO) and control WM793 cells treated with and without vemurafenib. (B) Quantification of invadopodia in STIM1 KO and control WM793 cells treated with and without vemurafenib (*n* = 3). (C) Quantification of STIM1 mRNA levels in vemurafenib-resistant and naïve WM793 treated with and without vemurafenib. (D) Effects of thapsigargin on Ca^2+^ influx in vemurafenib resistant and naïve WM793 cells. The results are representative of 3 replicates. (E) Western blot analyses of p-Pyk2 and STIM1 levels in control WM793 and vemurafenib-resistant WM793 cells treated with and without EGTA or W7. (F) Quantification of invadopodia in control WM793 and vemurafenib-resistant WM793 cells treated with and without EGTA or W7. ***P < 0.001.

Because PYK2 is regulated by intracellular Ca^2+^ and because STIM1 is an essential regulator of store-operated Ca^2+^ entry (SOCE) protein, we next investigated a possible link between these 2 proteins. We found that expressions of STIM1 both at the mRNA and protein levels were dramatically increased in vemurafenib-resistant melanoma cells (**Figure 6A and 6C**). Upon withdrawal of vemurafenib, STIM1 expression was reduced in vemurafenib resistant WM793 cells (**Figure 6C**). In addition, knockdown of STIM1 largely abrogated vemurafenib-induced PYK2 activation and invadopodia formation (**Figure 6A and 6B**). We further observed that the Ca^2+^ influx was dramatically increased in vemurafenib resistant cells when compared to naïve WM793 cells (**Figure 6D**). Together, these results indicated that vemurafenib induced PYK2 activation and invadopodia formation through STIM1.

We recently reported that SOCE regulated invadopodia formation through the Ca^2+^/CAM-PYK2 signaling axis^26^. To determine if vemurafenib treatment induced Pyk2 activation and invadopodia formation through Ca^2+^ and calmodulin, we treated cells with EGTA, a chelating agent for Ca^2+^, prior to assaying for p-PYK2 levels and invadopodia. We found that EGTA treatment dramatically reduced levels of p-PYK2 and the numbers of invadopodia (**Figure 6E and 6F**). Treatment with the calmodulin inhibitor, W7, also significantly inhibited PYK2 activation and invadopodia formation (**Figure 6E and 6F**). Overall, these results suggested that vemurafenib induced PYK2 activation and invadopodia formation through Ca^2+^ and calmodulin.

### Activation of PYK2 is associated with melanoma progression

**Figure 7:**
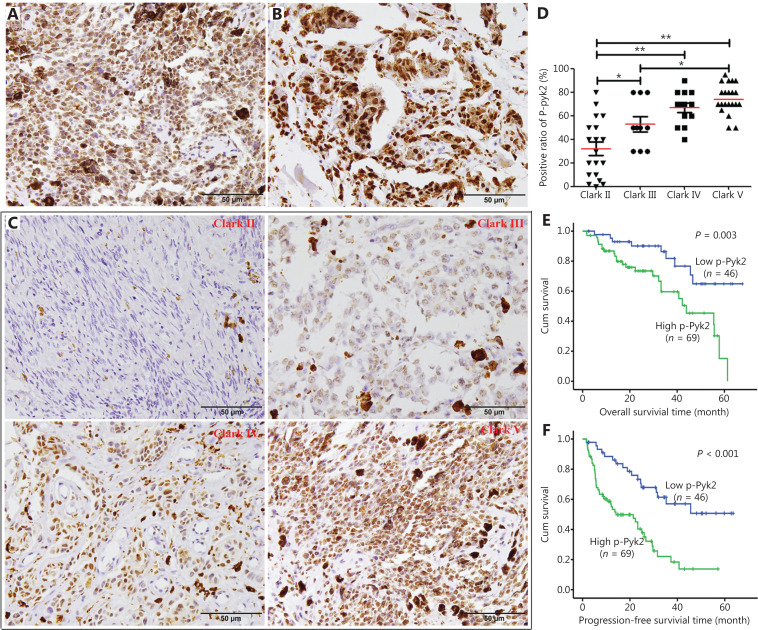
Expression of PYK2-Y402 in melanoma tissues and survival analysis of melanoma patients. (A, B) Representative images of p-PYK2 immunohistochemical staining in lymph node metastasis (A) and distant metastasis (B) of melanomas. (C) Representative images of p-PYK2 immunohistochemical staining of Clark grade II, III, IV, and V melanomas. (D) Comparison analyses of p-PYK2 expression levels in melanomas with different Clark grades. (E) The curves of overall survival according to p-PYK2 expressions in patients with melanomas. (F) The curves of progression-free survival according to p-PYK2 expressions in patients with melanomas. *P < 0.05; **P < 0.01.

Because there was no BRAF inhibitor treatment in our patient cohort, we could not determine the association between p-PYK2 levels and vemurafenib resistance in this study. However, we examined the levels of p-PYK2-Y402 in melanoma tissues and investigated the role of activation of PYK2 in melanoma progression. Among 180 melanoma tissues that were immunohistochemically stained with anti-p-PYK2 antibody, 133 cases could be evaluated. Among the 133 cases, 115 patients had complete clinicopathological and follow-up data (**Supplementary Tables S1 and S2**). The positive staining of p-PYK2 ranged from 0%–95%, with a median of 60%. We found that the expressions of p-PYK2 were significantly higher in patients with stage III and IV than those with stage I and II (*P =* 0.002) (**Supplementary Table S1**). There was no significant difference of p-PYK2 expressions between different groups based on sex, age or primary tumor sites (**Supplementary Table S1**). In cutaneous melanomas, the expression level of p-PYK2 was positively correlated with Clark grade (*P* < 0.001, *rs* = 0.663) (**Figure 7A, 7B, and 7D**). Together, these results indicated that activation of PYK2 was positively correlated with cell invasiveness (Clark grade) and tumor progression in melanomas.

We further evaluated the association of PYK2 activation with its prognosis. In this cohort, the median follow-up time was 24 months (2–68 months). Metastases were found in 62 patients (54.0%) at diagnoses or during the follow-up. The cases with metastases showed significantly higher levels of p-Pyk2 than those without metastasis (*P* < 0.001) (**Supplementary Table S1**). Patients with low p-PYK2 levels showed significantly longer OS and PFS than those with elevated levels of p-PYK2 (*P* < 0.001) (**Figure 7F**). Cox regression survival analysis showed that elevated p-PYK2 was an independent adverse predictor for OS [hazard ratio (HR) = 3.304, *P* = 0.003] (**Figure 7E**) and PFS (HR = 2.930, *P* < 0.001) (**Figure 7F**) in the 115 patients with melanomas (**Supplementary Table S2**). Taken together, these results indicated that activation of PYK2 correlated with melanoma progression and a poor prognosis.

## Discussion

We previously demonstrated that calcium signaling regulated invadopodia formation and cell invasion in melanomas by activation of the non-receptor tyrosine kinase Src^21^. In this study, we showed that PYK2 was activated by vemurafenib treatment and was required for vemurafenib-induced invadopodia formation and cell invasion in vemurafenib-resistant melanomas. Although focal adhesion kinase (FAK) and PYK2, 2 members of the FAK family, exhibit overlapping functions in focal adhesion formation^27^, we did not find an involvement of FAK in invadopodia formation in melanoma cells (**Supplementary Figure S2**). This is consistent with a previous report^20^. These findings suggested that PYK2 regulated tumor cell invasion and metastasis by controlling invadopodia formation, and that FAK controlled the invasiveness of tumor cells by regulating focal adhesion-mediated motility.

Consistent with *in vitro* results, we found that activation of PYK2 was correlated with melanoma invasiveness (Clark grade), advanced stage, and poor survival in patients with melanomas. COX survival analyses showed that p-PYK2 was an independent prognostic factor for OS and PFS of patients with melanoma. Therefore, our current study demonstrated the function of p-PYK2 in the progression of melanomas and also provided a potential prognostic marker for melanoma patients.

The present study also showed vemurafenib-induced invadopodia formation and cell invasion occurring by the activation of PYK2. Moreover, the PYK2 inhibitor, PF562271, overcame invadopodia formation and cell invasion in vemurafenib resistant melanoma cells. PF562271 has been shown to be a potential agent for the treatment of multiple myeloma^28^. Thus, our study also provided a potential treatment strategy, i.e., a combination of PYK2 inhibitor PF562271 with the BRAF inhibitor, vemurafenib, that may be an effective therapy to prolong the survival of patients with vemurafenib resistant melanomas.

## Conclusions

Overall, our current study showed that the STIM1-PYK2 axis mediated vemurafenib-induced formation of invadopodia and melanoma metastasis. In addition, phospho-PYK2 was a prognostic biomarker in patients with melanoma. A combination of PYK2 inhibitor with the BRAF inhibitor, vemurafenib, may therefore be an effective treatment for melanoma patients.
