# Supplementary material for: PYK2 mediates the BRAF inhibitor (vermurafenib)-induced invadopodia formation and metastasis in melanomas
Source: Cancer Biol Med. 2021 Sep 28;19(8):1211–23. doi: 10.20892/j.issn.2095-3941.2020.0294 (PMC9425182; doi:10.20892/j.issn.2095-3941.2020.0294)
Supplement: Supplementary file 1 [file cbm-19-1211-s001.pdf]

Supplementary materials

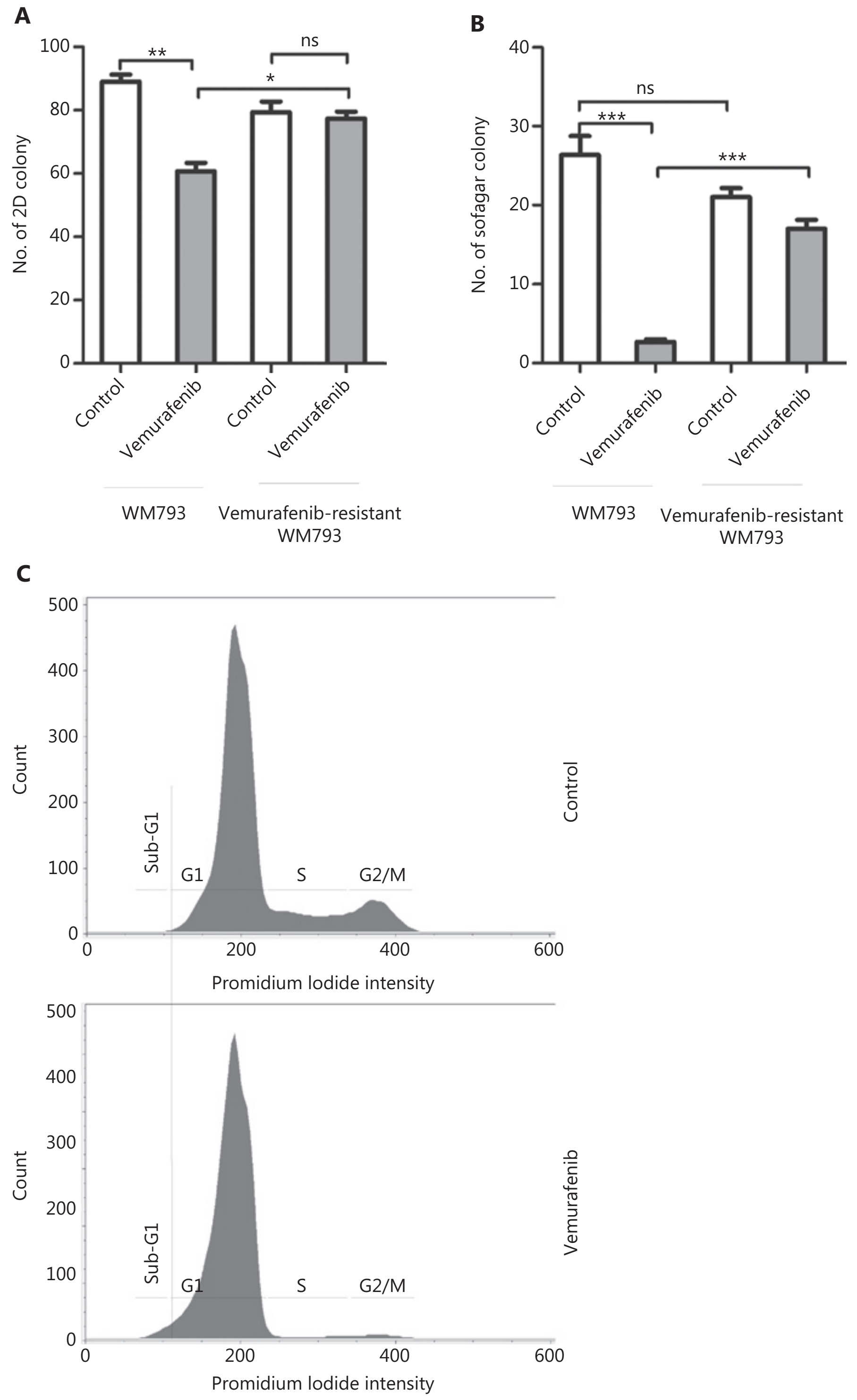

Figure S1 Effect of vemurafenib on WM793 cell growth. (A) The effects of vemurafenib on colony formation (two-dimensional culture) in naïve and vemurafenib resistant WM793 cells. (B) The effect of vemurafenib on colony formation (soft agar; three-dimensional cultures) in naïve and vemurafenib resistant WM793 cells. (C) The effects of vemurafenib on cell cycle arrest and apoptosis in naïve WM793 cells. *$P < 0.05$; **$P < 0.01$; ***$P < 0.001$.

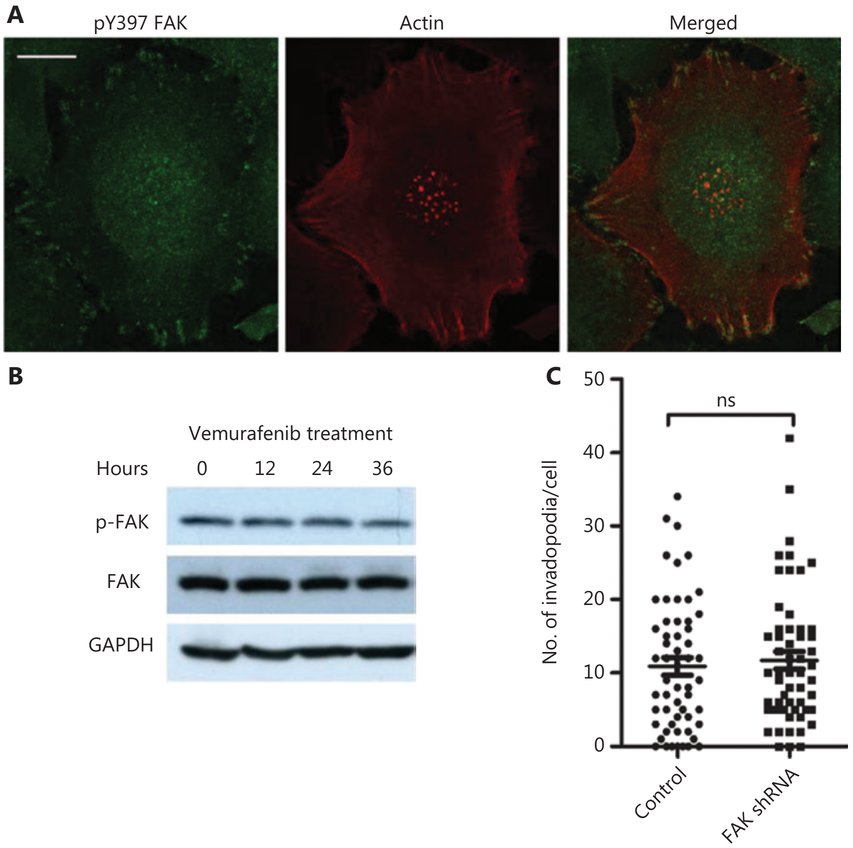

Figure S2 FAK is not activated and is not involved in invadopodia formation. (A) Immunofluorescence staining of p-FAK and invadopodia (actin staining). Scale bars = 10 μm. (B) Western blot analysis showed that vemurafenib treatment did not affect FAK or p-FAK levels. (C) The effect of FAK shRNA on invadopodia formation. PYK2, proline-rich tyrosine kinase 2; FAK, focal adhesion kinase; DTIC, dacarbazin; TMA, tissue microarray; OS, overall survival; PFS, progression-free survival; PBS, phosphate-buffered saline; ECM, extracellular matrix.

Table S1 The relationships between p-Pyk2 expressions and clinicopathological parameters in 115 patients with melanomas

Parameters	*n*	Mean ± SEM	*P*
Sex			
Male	69	57.5 ± 3.0	0.285
Female	46	52.2 ± 4.1	
Age (years old)			
< 60	60	56.0 ± 3.7	0.780
≥ 60	55	54.7 ± 3.2	
Tumor site			
Acra	86	54.8 ± 2.8	0.696
Non-acra*	29	57.0 ± 5.3	
Stage			
I and II	81	50.5 ± 3.1	0.002
III and IV	34	66.9 ± 3.1	
Metastasis**			
No	53	44.2 ± 4.0	< 0.001
Yes	62	64.2 ± 2.5	

*Includes non-acral cutaneous and mucosal melanomas.
**Includes metastasis at diagnosis and during the follow-up.

Table S2 The COX regression analyses of overall survival and progression-free survival in 115 patients with melanomas

Parameters	OS			PFS		
	P	RR	95% CI for RR	*P*	RR	95% CI for RR
Sex (female *vs*. male)	0.805	1.093	0.537–2.224	0.438	1.247	0.714–2.175
Age (≥ 60 *vs*. < 60 years old)	0.470	1.297	0.640–2.628	0.844	1.058	0.602–1.861
Tumor site (non-acra *vs*. acra)	0.001	3.406	1.601–7.247	0.078	1.740	0.939–3.224
Stage (III/IV *vs*. I/II)	0.008	2.573	1.284–5.153	0.033	1.879	1.052–3.354
p-Pyk2 (high *vs*. low)	0.007	3.304	1.385–7.883	0.001	2.930	1.518–5.657
DTIC treatment (no *vs*. yes)	0.576	0.794	0.354–1.783	0.716	1.129	0.588–2.168
Biotherapy* (no *vs*. yes)	0.788	0.883	0.355–2.194	0.808	1.094	0.530–2.258

*Includes treatments with IFN, IL-2, LAK, and tumor vaccine. OS, overall survival; PFS, progression-free survival; DTIC, dacarbazine.
